# Influence of Short-Term Fasting on the Efficacy of Albendazole Against Benzimidazole-Resistant *Haemonchus contortus* Under Farm Conditions

**DOI:** 10.3390/vetsci13060540

**Published:** 2026-05-30

**Authors:** Michal Babják, Alžbeta Königová, Tetiana A. Kuzmina, Ľudmila Burcáková, Marián Várady

**Affiliations:** 1 Institute of Parasitology, Slovak Academy of Sciences, Hlinkova 3, 040 01 Košice, Slovakia; babjak@saske.sk (M.B.); konig@saske.sk (A.K.); takuzmina@gmail.com (T.A.K.); burcakova@saske.sk (Ľ.B.); 2 I. I. Schmalhausen Institute of Zoology NAS of Ukraine, Bogdan Khmelnytsky Street 15, 01030 Kyiv, Ukraine

**Keywords:** fasting, anthelmintic resistance, faecal egg count reduction test, effective dose, gastrointestinal nematodes, goats, *Haemonchus contortus*

## Abstract

The aim of this study was to evaluate the effect of short-term dietary restriction on the in vivo efficacy of albendazole (ALB) on a goat farm with suspected reduced efficacy of benzidazoles (BZs). Initially, pooled faecal samples were analysed using the in vitro egg hatch test (EHT) to assess the level of BZ resistance. Subsequently, 58 goats were allocated into six experimental groups: three groups received a single dose of ALB (5 mg/kg body weight, BW) and three groups received a double dose (10 mg/kg BW). Each group was subdivided into animals fasted for 24 h before treatment, 24 h after treatment, and non-fasted controls. Morphological identification of third-stage larvae before and after treatment confirmed the predominance of *Haemonchus contortus* (91% and 100%, respectively). The in vitro EHT demonstrated reduced susceptibility to BZs, with the concentration of thiabendazole (TBZ) required to inhibit 50% of egg hatching exceeding the resistance threshold of 0.05 μg/mL, ranging from 0.166 to 0.247 μg/mL TBZ. Despite the presence of BZ resistance, fasting significantly increased the in vivo efficacy of ALB by up to 18%. These findings demonstrate that short-term dietary manipulation represents an effective strategy to enhance anthelmintic efficacy and partially overcome reduced drug performance.

## Simple Summary

Gastrointestinal parasites cause health problems and significant production losses on small ruminant farms worldwide. One of the major issues is the ability of these parasites to develop resistance to commonly used anthelmintic drugs. In this study, we attempted to influence the efficacy of albendazole by fasting the animals for 24 h and increasing the recommended dose on a goat farm with established resistance to benzimidazoles. The most significant increase in efficacy was observed in the group of goats fasted for 24 h after administration of the drug. These findings indicate that optimizing the drug dosage and improving its bioavailability in the host through controlled fasting may offer a practical strategy for managing parasite populations on farms with established anthelmintic resistance.

## 1. Introduction

Among the all broad-spectrum anthelmintic groups available for the treatment of grazing animals to control parasitic nematodes (benzimidazoles, imidazothiazoles/hydropyrimidines, and macrocyclic lactones) [1], the benzimidazole (BZ) anthelmintic albendazole (ALB) remains the most widely used drug on small ruminant farms due to its low price, high market availability, and broad-spectrum efficacy with low toxicity [2,3,4]. However, because of frequent treatments and improper dosing, ALB also has one of the highest incidences of anthelmintic resistance (AR) cases in parasitic nematodes on sheep and goat farms worldwide [5,6,7,8,9]. With the emergence of AR and the spread of resistant parasites on small ruminant farms, there are very few ways to maintain production and good animal health [10,11,12]. At present, alternative treatment methods, such as the use of herbal nutraceuticals and bioactive plants, are increasingly adopted for their antioxidant, antimicrobial, anti-inflammatory, anticoccidial, and anthelmintic properties. These compounds primarily act by stimulating the host’s immune system, improving digestion, and enhancing the quality of milk and meat production in sheep and goats, but they still cannot fully replace traditional chemical treatments for helminth parasites.

Stimulating the host immune system is also possible with vaccines (Barbervax^®^) against the most pathogenic gastrointestinal nematode (GIN), *Haemonchus contortus* [13]. However, these vaccines have been licensed for commercial use only in Australia and are also available in a few other countries, including the United Kingdom and South Africa [13,14]. New broad-spectrum active substances are unavailable on the local market; therefore, chemical-based treatment of small ruminants remains limited to three main groups of anthelmintics (BZs, imidazothiazoles/hydropyrimidines and macrocyclic lactones). For this reason, various approaches are being sought to improve the efficacy of anthelmintics. The basic principle of these methods is to modulate and increase the bioavailability of the drug’s active substance in the host organism [15]. In the case of ALB, the primary active compound in the plasma is albendazole–sulphoxide (ALB–SO) and less active albendazole sulfone (ALB–SO2) [16,17].

The efficacy of active compounds is influenced by the duration of the parasite’s exposure to an effective concentration of the drug within the body of the host [18]. The commonly used method was described by Sanyal [19], who significantly increased the plasma bioavailability of the recommended dose of ALB by dividing its administration into a two-step application. The efficacy and bioavailability of ALB can also be improved by increasing the effective dose [20,21]. This approach is particularly important in goats, as it has long been known that goats have a different metabolic rate from sheep and can metabolise the active compounds of the drug faster [22,23,24]. Another challenge for goat farms is the relatively small number of anthelmintics officially licensed for use on goats. As a result, treatment schemes designed for sheep are often applied to goats, leading to underdosing of anthelmintics and the development of AR [24,25].

Another widely used method to increase the anthelmintic efficacy is based on fasting or reduced feed intake in animals prior to their treatment, which decreases the gastrointestinal passage rate of digesta and, subsequently, enhances the absorption and bioavailability of the drug [26,27,28,29]. The positive effect of fasting has also been described in sheep treated with ALB, where prolonged persistence and increased concentrations of ALB–SO and ALB–SO2 were observed in plasma and tissues after reducing feed intake prior to treatment [30]. The increased concentrations of both active ALB compounds were measured in the gastrointestinal mucosa and fluids of fasted calves compared to those without feed restriction [31]. However, very few field studies have examined the effects of short-term fasting on the GIN of small ruminants [11], especially in goats.

The primary objective of this study was to evaluate the effect of short-term dietary modification, specifically 24-h fasting applied either before or after treatment, on the in vivo efficacy of ALB administered at single and double doses in goats naturally infected with gastrointestinal parasites. In this context, we aimed to determine whether short-term fasting could serve as a practical strategy to modulate and enhance the efficacy of ALB treatment in goats raised on a farm exhibiting clinical signs of haemonchosis and suspected BZ resistance.

## 2. Materials and Methods

### 2.1. Trial Design

The study was conducted in October–November 2025 on a goat farm located in the Central region of Slovakia, and the herd consisted of approximately 300 Anglo-Nubian goats reared mainly for milk production. The animals were managed under an extensive grazing system with an ecological pasture-based management approach. Goats were grazed on natural pastures from April to October and were housed during the winter period. Nutrition during the grazing season was based primarily on pasture vegetation, with supplementary feeding provided when necessary according to production requirements. Routine antiparasitic treatment was performed twice annually as part of the herd health management program. On this farm, frequent deaths of goat kids and clinical signs of haemonchosis, such as anaemia, submandibular oedema and poor body condition, were registered. Also, the farm had a history of long-term use of BZ anthelmintics. Fifty-eight female goats aged between 1 and 3 years were selected for the study. All selected goats had not been treated with any anthelmintics for at least 10 weeks prior to the start of the experiment.

The study was divided into an in vitro (phase 1) and an in vivo (phase 2) part.

### 2.2. Phase 1—Egg Hatch Test

In phase 1, pooled faecal samples were collected from all 58 goats selected for the study. Strongyle-type eggs from *Trichostrongylidae* and *Chabertiidae* family were isolated from anaerobically stored pooled samples and used for the in vitro egg hatch test (EHT) performed according to the description of Várady et al. [32]. The suspension of eggs was incubated in 24-well plates with different concentrations (0.05, 0.1, 0.3, 0.5 and 1 μg/mL) of thiabendazole (TBZ, Merck, Darmstadt, Germany). The EHT was performed four times (trials ##1–4), with two replicates per TBZ concentration. The proportion of unhatched and hatched eggs was recorded for each concentration using a Leica DM IL inverted microscope (Leica Microsystems, Wetzlar, Germany) at 40× magnification.

### 2.3. Phase 2—Faecal Egg Count Reduction Test

In the in vivo phase 2, the 58 selected goats were divided into six experimental groups of 9–10 individuals as follows:

− Group 1 (G1; n = 10) was treated with a single dose (5 mg/kg BW) of ALB, without fasting.
− Group 2 (G2; n = 10) was treated with a single dose (5 mg/kg body weight, BW) of ALB (Albendavet, Divasa-Farmavic, Barcelona, Spain); the goats were not fed for 24 h before treatment.
− Group 3 (G3; n = 10) was treated with a single dose (5 mg/kg BW) of ALB; the goats were not fed for 24 h after treatment.
− Group 4 (G4; n = 9) was treated with a double dose (10 mg/kg BW) of ALB without fasting.
− Group 5 (G5; n = 9) was treated with a double dose (10 mg/kg BW) of ALB; the goats were not fed for 24 h before treatment.
− Group 6 (G6; n = 10) was treated with a double dose (10 mg/kg BW) of ALB; the goats were not fed for 24 h after treatment.

Only clinically healthy adult goats with good body condition and continuous access to drinking water were enrolled in the study; informed owner consent was obtained for a short-term (24 h) feed withdrawal before and/or after drug administration. During the 24-h fasting period, the animals were continuously monitored under the supervision of the animal owner and a veterinarian. Goats were weighed before ALB administration using a portable scale; the dose for each goat was calculated individually based on its weight.

Faecal samples from each goat were collected individually and directly from the rectum on the day of treatment (Day 0) and 10 days after treatment (Day 10). The level of goat infection with gastrointestinal nematodes was determined for each goat individually using the modified McMaster method with a sugar solution (specific gravity of 1.28) and a sensitivity of 50 eggs per gram (EPG) [1,33].

### 2.4. Faecal Egg Count Reduction Test (FECRT)

The FECRT was based on individual evaluation of efficacies [34] and was calculated using the following formula:

FECR = (1/n) ∑ (100 × (1 − [Ti2/Ti1])

where Ti2 is the post-treatment and Ti1 is the pre-treatment EPG in host i, from a total of n hosts.

### 2.5. Morphological Differentiation of Third-Stage (L3) Larvae

To obtain the infective third-stage (L3) larvae, coprocultures were prepared from a pooled sample of G1–G6 collected on Day 0, and from each treated goat group collected on Day 10. For this, faecal samples were thoroughly mixed with about 10% tap water, placed in a plastic container, and incubated in a thermostat at +27 °C for 7 days. After incubation, L3 were harvested using the Baermann technique. From each coprocultured sample, 100 randomly selected L3 were stained with iodine and identified by morphological features and assigned to the species/genus level as described by Van Vyk and Mayhew [35].

### 2.6. Statistical Analysis

The EHT data were analysed using a logistic regression model [36] to determine the effective concentrations of TBZ required to prevent 50% (effective dose ED_50_) and 99% (effective dose ED_99_) hatching of eggs. Drug efficacy, expressed as a percentage, was compared among groups using one-way analysis of variance ANOVA (GraphPad Software. GraphPad Prism. Version 9.0. (San Diego, CA, USA): GraphPad Software; 2023), with statistical significance set at *p* < 0.05; post hoc comparisons were performed using Dunnett’s test, with the non-feed-restricted group serving as the control, to evaluate the effect of feed restriction on drug efficacy.

## 3. Results

### 3.1. Phase 1—EHT

The EHT conducted at the goat farm indicated the presence of BZ-resistant nematodes; mean egg hatching percentages at the monitored concentrations of 0.1 and 0.3 μg/mL TBZ ranged from 61.25% to 68.70% and from 23.65% to 38.90%, respectively (Table 1). ED_50_ values exceeded the threshold of 0.05 μg/mL TBZ in all four assays, ranging from 0.166 μg/mL to 0.247 μg/mL TBZ; ED_99_ values ranged from 4.279 μg/mL to 6.418 μg/mL TBZ.

### 3.2. Phase 2—FECRT

On Day 0, the individual EPG levels of strongyle-type eggs from Trichostrongylidae and Chabertiidae family of all tested groups ranged from 250 to 14,850. The average group’s EPG level recorded on Day 0 ranged from 715.0 ± 560.8 in group G6 to 4480.0 ± 4098.0 in group G2 (Table 2). Eggs of *Nematodirus* spp., *Moniezia* spp., and *Trichuris* spp. were also detected; the EPG levels for these parasites were not estimated. On Day 10, only Trichostrongylidae and Chabertiidae family strongyle-type eggs were detected in goats across all tested groups; average EPG levels ranged from 105.0 ± 136.8 in group G6 to 2305.0 ± 2920.0 in group G2.

According to the results of FECRT, all tested groups were evaluated as having “reduced” ALB efficacy (Table 2). The lowest ALB efficacies (54.14% and 56.16%) were observed in the group that fasted for 24 h before treatment with a single dose of ALB, and in the group treated with a single dose of ALB without fasting, respectively. The highest ALB efficacy was observed in group G6, which received a double dose of ALB and fasted for 24 h after treatment. At both ALB doses (5 mg and 10 mg), drug efficacy differed significantly among groups (*p* < 0.05 and *p* < 0.01, respectively). Post hoc analysis using Dunnett’s test demonstrated that, at the 10 mg dose, significant differences in efficacy were observed in the feed-restricted group subjected to pre-treatment fasting (*p* = 0.0372) as well as in the feed-restricted group subjected to post-treatment fasting (*p* = 0.0068).

### 3.3. Morphological Differentiation of L3 Larvae

Morphological identification of infective larvae in coprocultures prepared from pooled (G1–G6, mixed) samples before ALB treatment (Day 0) revealed the presence of two nematode species: *Haemonchus contortus* (91% of L3 collected) and *Teladorsagia circumcincta* (9%). On Day 10 after ALB treatment, *H. contortus* was the only nematode species identified in all six (G1–G6) groups of goats.

## 4. Discussion

The present study was conducted on a goat farm where clinical signs consistent with haemonchosis were frequently observed, including severe anaemia, pale mucous membranes, reduced growth, weight loss, weakness, and occasional sudden death of young animals. According to the farm owners, goats had been routinely treated with BZ anthelmintics; however, the efficacy of these treatments had never been formally evaluated. Morphological identification of infective larvae (L3) in pooled faecal samples confirmed the predominance of *Haemonchus contortus*, which accounted for 91% of recovered larvae prior to treatment. The high prevalence of *H. contortus*, together with the presence of *Nematodirus* spp. and *Moniezia* spp. eggs detected in faecal samples before treatment, likely contributed to the elevated mortality of goat kids observed on the farm. Consequently, the first phase of the study focused on assessing the presence of BZ resistance in the *H. contortus* population using the in vitro egg hatch test (EHT).

The EHT can be interpreted either by calculating the ED_50_ and ED_99_ threshold values or by evaluating the proportion of eggs hatching at specific drug concentrations. The ED_50_ threshold is generally set at 0.1 μg/mL thiabendazole (TBZ) [1], although a lower threshold of 0.05 μg/mL TBZ has been recommended when *H. contortus* is the dominant species [37]. In the present study, ED_50_ values obtained in all four EHT trials exceeded the recommended threshold. Similarly, ED_99_ values considered a more sensitive indicator of BZ resistance [38], substantially exceeded the recommended limit of 0.3 μg/mL TBZ. Previous studies have suggested that monitoring the proportion of egg hatching at 0.1 and 0.3 μg/mL TBZ provides a more sensitive indicator than ED_50_ or ED_99_ values and may serve as a reliable predictor of the frequency of resistant alleles in *H. contortus* populations [39,40], as well as of the expected in vivo efficacy of BZ anthelmintics assessed by the faecal egg count reduction test (FECRT) [7,11,41].

Based on the mean proportion of eggs hatching at 0.1 μg/mL TBZ (61.25–68.70%) and 0.3 μg/mL TBZ (23.65–38.90%), the expected in vivo efficacy of ALB was estimated to range between 40% and 60% of reduction [39]. This prediction corresponded well with the observed efficacy in two groups (G1 and G2) treated with a single dose of ALB, irrespective of fasting status. However, in the remaining groups, the in vivo efficacy exceeded the values predicted by the in vitro EHT. One explanation for these discrepancies may lie in the genetic structure of the parasite population. The efficacy of ALB can be influenced by the relative proportion of susceptible, heterozygous-resistant, and homozygous-resistant alleles present in the nematode population, as previously demonstrated in experimental studies using *H. contortus* as a model parasite [42].

Because *H. contortus* accounted for the vast majority of infective larvae on the farm, the conditions of the present study resembled those of experimental monoinfections rather than the mixed gastrointestinal nematode infections typically encountered under field conditions. Under such circumstances, it can be assumed that egg hatching at the monitored EHT concentrations, as well as the efficacy of ALB in single-dose groups, primarily reflected the susceptibility of the sensitive fraction of the parasite population, including *H. contortus* and *Teladorsagia circumcincta* (9%). In contrast, treatment with a double dose of ALB likely affected not only susceptible individuals but also a proportion of heterozygous worms carrying resistant alleles. At the same time, approximately 15% of *H. contortus* individuals that remained unaffected even after administration of a double dose of ALB were probably homozygous for resistance-associated alleles.

A key finding of the present study was the positive effect of short-term fasting on the in vivo efficacy of ALB. Across the tested treatment regimens, 24-h dietary restriction increased the efficacy of ALB treatment by up to 18% compared with non-fasted control groups. This improvement was particularly evident in the group fasted for 24 h after administration of a single dose of ALB (G3), where treatment efficacy was approximately 20% higher than in the other two single-dose groups (G1 and G2). Similarly, in goats treated with a double dose of ALB, both fasting regimens (before or after treatment) resulted in higher efficacy compared with the non-fasted double-dose group (G4), which showed approximately 12–16% lower efficacy. These results suggest that short-term fasting can enhance the therapeutic performance of ALB even in parasite populations with confirmed BZ resistance.

Nevertheless, it should be noted that the groups fasted after treatment had slightly lower mean faecal egg counts at the start of the experiment despite efforts to distribute animals with different body conditions and infection intensities evenly among groups. Differences in baseline EPG values between groups are relatively common in naturally infected animals due to the heterogeneous distribution of parasite burdens. In addition, the 24-h fasting protocol did not negatively affect animal body weight or general clinical condition. A more precise evaluation of the relative effectiveness of the six treatment approaches would therefore require a pharmacokinetic study examining plasma concentrations of ALB and its active metabolites. Several pharmacokinetic studies have demonstrated that fasting can increase drug availability and prolong systemic exposure. For example, Sanchez et al. [31] reported increased plasma availability of ALB (37–118%) and prolonged peak concentrations in fasted calves compared with fed animals, together with increased concentrations of active metabolites in target tissues and fluids. Similarly, Hennessy et al. [30] observed significantly higher plasma concentrations of albendazole sulfoxide (ALB-SO) 24 h after treatment in fasted sheep, accompanied by approximately 20% increased efficacy against *H. contortus* and *Trichostrongylus colubriformis*. Alvarez et al. [43] also reported increased plasma availability of ALB in fasted sheep; however, this did not translate into differences in in vivo efficacy between fasted (49%) and fed animals (48%), which is partly consistent with our observations in two of the three single-dose groups. In contrast, Karademir et al. [44] reported significantly higher plasma concentrations of ALB-SO and ALB-SO_2_ in fed goats compared with fasted goats, suggesting that goats may differ from other ruminant species in the metabolism of ALB. Although fasting-associated increases in the bioavailability and systemic exposure of ALB metabolites have been documented in ruminants, corresponding changes in elimination half-life (T½) have not been consistently reported. Therefore, the improved efficacy observed in the present study may be more closely associated with enhanced drug availability and prolonged exposure at the target site rather than with a direct alteration of elimination kinetics. Considering that this study was conducted on a single farm and included only female goats of one breed, without accompanying pharmacokinetic analyses, the findings should be interpreted with appropriate caution. Further studies under field conditions are therefore needed to confirm these observations while taking these limiting factors into account.

An important implication of the present findings is that short-term dietary modulation achieved an increase in ALB efficacy that was comparable to that obtained by doubling the administered dose of the anthelmintic. In our experimental conditions, 24-h fasting enhanced treatment efficacy by up to 18%, representing an effect similar in magnitude to a 100% increase in drug dosage. From a practical and environmental perspective, this observation is highly relevant. Increasing the dose of anthelmintics is commonly associated with elevated drug residues in animal products and enhanced excretion of active metabolites into the environment, which may negatively affect non-target organisms and contribute to ecological contamination. In contrast, nutritional manipulation represents a non-pharmacological strategy that can improve drug performance without increasing chemical input. Therefore, the application of short-term fasting may offer a sustainable alternative to dose escalation, potentially reducing environmental burden while maintaining or even enhancing therapeutic efficacy. These findings suggest that both short-term fasting and appropriate dosing strategies may serve as useful tools for modulating anthelmintic efficacy in field conditions. However, these measures should be integrated into comprehensive parasite control programs, including regular monitoring of drug efficacy and improved farm management practices, in order to prevent the further development of highly resistant gastrointestinal nematode populations.

## Figures and Tables

**Table 1:** Results of the in vitro egg hatch test (EHT) performed on the goat farm: Mean hatching in % (with SD), and effective doses ED_50_ and ED_99_ for each of four trials (# 1–4).

Concentration of TBZ ^1^, μg/mL	Mean Hatching ± SD ^2^ (%)			
	Trial#1	Trial #2	Trial #3	Trial #4
Control	91.25 ± 1.55	88.30 ± 1.40	87.60 ± 0.20	91.80 ± 0.90
0.05	84.35 ± 2.25	82.45 ± 1.35	84.35 ± 1.25	80.50 ± 0.70
0.1	61.25 ± 1.55	68.35 ± 0.45	66.10 ± 2.40	68.70 ± 1.60
0.3	23.65 ± 1.85	38.90 ± 0.80	36.85 ± 3.75	28.65 ± 1.15
0.5	19.15 ± 0.35	23.60 ± 2.70	22.00 ± 4.90	22.05 ± 1.75
1.0	12.45 ± 0.25	11.55 ± 0.45	12.90 ± 1.10	10.55 ± 0.35
	Effective TBZ concentrations			
ED_50_ ^3^	0.166	0.247	0.232	0.195
ED_99_ ^4^	4.279	6.418	6.322	5.126

^1^ Thiabendazole; ^2^ Standard deviation (±); ^3–4^ Effective concentrations of TBZ to prevent 50% and 99% hatching of eggs.

**Table 2:** Results of the in vivo faecal egg count reduction tests (FECRT) performed on the goat farm.

Group of Goats/Treatment Regimes	Mean EPG ^1^ ± SD ^2^		FECR (%) ± SD
	Day 0 ^5^	Day 10 ^6^	
G1 (n ^7^ = 10)/ ALB ^3^ (5 mg/kg BW ^4^); without fasting	2410.0 ± 1298.6	1075.0 ± 622.6	56.16 ± 7.74
G2 (n = 10)/ ALB (5 mg/kg BW); fasting before treatment	4480.0 ± 4098.0	2305.0 ± 2920.0	54.14 ± 20.21
G3 (n = 10)/ ALB (5 mg/kg BW); fasting after treatment	740.0 ± 377.4	180.0 ± 155.2	74.74 ± 24.08
		*p*-value	0.0483
G4 (n = 9)/ ALB (10 mg/kg BW); without fasting	3666.7 ± 1476.3	988.9 ± 435.1	72.79
G5 (n = 9)/ ALB (10 mg/kg BW); fasting before treatment	2883.3 ± 4362.7	488.9 ± 849.8	84.89 ^a^
G6 (n = 10)/ ALB (10 mg/kg BW); fasting after treatment	715.0 ± 560.8	105.0 ± 136.8	88.45 ^b^
		*p*-value	0.0099

^1^ Eggs per gram; ^2^ Standard deviation; ^3^ Albendazole; ^4^ Body weight; ^5^ Day of treatment; ^6^ 10 days after treatment; ^7^ number of goats. Different letters within a column indicate significant differences in FECR between non-fasted control and treated groups at *p* < 0.05.

## Data Availability

The original contributions presented in this study are included in the article. Further inquiries can be directed to the corresponding author.
